# Changes in Morphology and Activity of Transglutaminase Following Cross-Linking and Immobilization on a Polypropylene Microporous Membrane

**DOI:** 10.3390/molecules161210046

**Published:** 2011-12-05

**Authors:** Yan-Guo Shi, Lei Qian, Na Zhang, Chun-Ran Han, Ying Liu, Yi-Fang Zhang, Yong-Qiang Ma

**Affiliations:** 1 Key Laboratory of Food Science and Engineering of Heilongjiang Province, Harbin University of Commerce, Harbin 150076, China; 2 Harbin Hi-tech Soybean Food Co. Ltd., Harbin 150078, China

**Keywords:** transglutaminase, cross-linking, immobilization, enzyme activity, microstructure

## Abstract

Transglutaminase (TGase) was cross-linked with glutaraldehyde, and cross-linked crystalline transglutaminase was immobilized on a polypropylene microporous membrane by UV-induced grafting. Immobilized enzyme activity were calculated to be 0.128 U/cm^2^ polypropylene microporous membrane. The microstructure and enzyme characteristics of free, cross-linked and immobilized transglutaminase were compared. The optimum temperature of free transglutaminase was determined to be approximately 40 °C, while cross-linking and immobilization resulted in an increase to approximately 45 °C and 50 °C. At 60 °C, immobilized, cross-linked and free transglutaminase retained 91.7 ± 1.20%, 63.2 ± 1.05% and 37.9 ± 0.98% maximum activity, respectively. The optimum pH was unaffected by the state of transglutaminase. However, the thermal and pH stabilities of cross-linked and immobilized transglutaminase were shown to increase.

## Abbreviations

(GD): grafting degree
(SEM): scanning electron microscopy
(SD): standard deviation
(TCA): trichloracetic acid
(TGase): transglutaminase

## 1. Introduction

Transglutaminase (TGase; protein-glutamine-glutamyltransferase, EC 2.3.2.13). catalyzes the acyl-transfer reaction in which the γ-carboxyamide groups of glutamine residues in proteins, peptides and various primary amines, act as acyl donors and primary amino groups including ε-amino groups of lysine residues, either as peptide-proteins bound or free lysine, act as the acyl acceptors [1,2,3,4,5]. These reactions are dependent on a number of factors, including pH and temperature. Transglutaminases present in most animal tissues and body ﬂuids are involved in several biological processes, including blood clotting, wound healing, epidermal keratinization, and stiffening of the erythrocyte membrane [6].

Intra- and intermolecular cross-linking as well as the addition of peptide or other moieties to proteins has the potential for causing desirable changes in functionality [7]. For example, transglutaminase has been used to form gels [8,9,10,11,12], to form cross-linked hompolymers or heteropolymera of proteins [13,14,15,16,17,18], and to introduce other moieties into the protein structure [19,20]. Industrially, TGase is mainly used to improve the texture, stability, and other functional properties of food products. In the meat industry, using transglutaminase to cross-link proteins has been found feasible. The TGase enzyme catalyzes cross-linking between protein molecules [21]. The TGase currently utilized has a microbiological origin, which has been tested in the manufacture of several meat and ﬁsh products [22]. Kuraishi *et al.* [23] have developed the new meat-binding system using TGase and caseinate simultaneously. Seki *et al.* [24] found that endogenous fish TGase caused ‘suwari’ setting, hardening fish protein paste at low temperature through crosslinking. In another study, Moore *et al.* [25] reported that gluten-free batter (prepared from rice flour, potato starch, corn flour and xanthan gum) enriched with skim milk powder or egg white and treated with TGase promoted covalent bond formation between lysine and glutamine residues in the proteins and improved the protein network of the batter. TGase from walleye pollack, fish for surimi, has been purified and characterized by Kumazawa *et al.* [26]. Otherwise, many researchers have shown that TGase can be used in the processing of dairy, wheat and soybean products in order to improve texture, water-holding capacity, elasticity, nutritional value and appearance [27,28,29,30,31,32,33,34].

Enzyme stabilization [35] is one of the major challenges in the biocatalytic process optimization. Enzymes have to be used at higher temperatures, shear rate and organic solvent environments for the production [36] of pharmaceuticals, agrochemicals, consumer care products, *etc.* Cross-linked enzyme crystals technology [37] is one of the most exciting developments in the area of biocatalysis. Cross-linked enzyme crystals are prepared by controlled precipitation of enzymes into microcrystals followed by cross-linking using bifunctional reagents to form strong covalent bond between free amino acid groups in the enzyme molecules [38]. In a cross-linked enzyme crystal, the lattice interactions in the enzyme crystal when fixed by inter- and intramolecular chemical cross-links provide additional physical and thermal stability. A protein in cross-linked crystal is stabilized by links in all three-dimensional structure. Hence, cross-linked enzyme crystals are highly active, recyclable and having good mechanical stability.

Immobilization of enzymes is one of the techniques used by the industries to bring down the cost of the process by reusing the enzymes. Sangha [39] examine the possibility of using an immobilized form of transglutaminase for cross-linking of proteins. Transglutaminase was covalently immobilized on poly(lysy1)-α-casein which was covalently attached to 3-aminopropyl porous glass. This represents the first study of a covalently immobilized form of this enzyme. However, information regarding the microstructure and enzyme characteristics of immobilized transglutaminase is limited.

In this study, transglutaminase was cross-linked and immobilized on a polypropylene microporous membrane to improve the enzyme characteristics. The surface of polypropylene microporous membranes was activated by UV-induced graft polymerization of methyl methacrylate to improve the poor biocompatibility of this membrane and activity of the enzyme. Scanning electron microscopy (SEM) was used to study the microstructure of the free, cross-linked and immobilized crystalline transglutaminase. The optimal temperature and pH conditions for transglutaminase activity were determined in addition to analysis of thermal and pH stabilities.

## 2. Results and Discussion

Units of enzyme activity immobilized on spacers were calculated to be 0.128 U/cm^2^ polypropylene microporous membrane. The activity of immobilized enzyme was measured using the hydroxamate method relative to the total amount of protein estimated by the Bradford assay and was calculated to be 31.2 U/mg, which was lower than that of free enzyme (102.8 U/mg). It can be speculated that the loading capacity and activity of the immobilized enzyme is attributed to the surface properties of the polypropylene microporous membrane of polymeric spacers such as coarseness and hydrophilicity.

### 2.1. SEM Investigations

**Figure 1:**
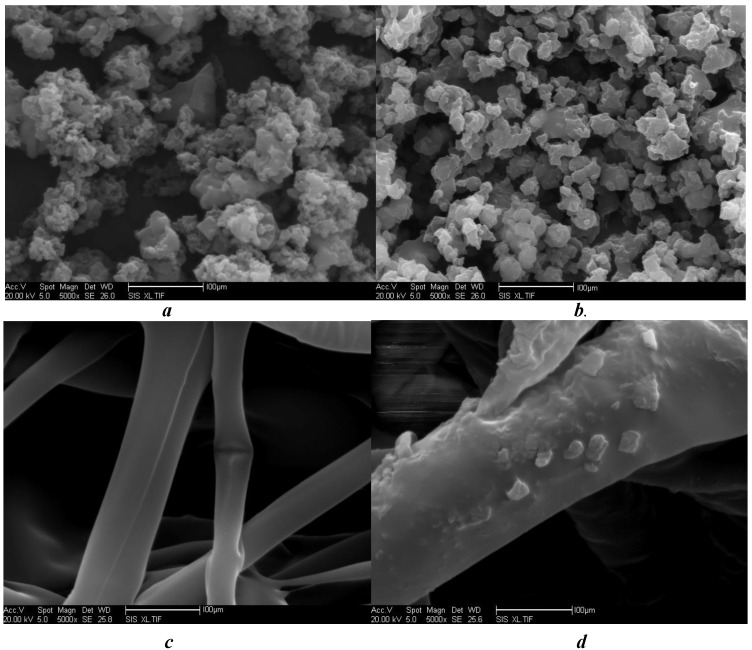
SEM photomicrographs of free transglutaminase (**a**) cross-linked transglutaminase (**b**), polypropylene microporous membrane in the absence of (**c**) and with (**d**) the cross-linked transglutaminase (magnification ×5,000).

Surface characteristics and cross-sectional morphology of free, cross-linked and immobilized transglutaminase and polypropylene microporous membranes were analyzed by SEM (Figure 1).

The size of free transglutaminase was not uniform, and the shape was irregular (Figure 1a). However, the particle size of cross-linked transglutaminase was greater and the boundaries more distinct. Particle shape was that of an irregular polyhedron (Figure 1b). Furthermore, the spatial molecular configuration of cross-linked transglutaminase was altered from that of the free enzyme. The surface fibers of the native polypropylene microporous membrane were relatively smooth with an irregular, crossed arrangement (Figure 1c). However, after immobilization, the surface of fibers of the polypropylene microporous membrane were more coarse, with particles of uniform size adhered to the surface (Figure 1d). The density of these particles on the fiber surface was high and furthermore, the form of the particles was similar to that of cross-linked transglutaminase (Figure 1b). These observations confirmed immobilization of cross-linked transglutaminase onto the surface of the polypropylene microporous membrane.

### 2.2. Optimal Temperature and pH

The influence of temperature and pH on enyme activity of cross-linked and immobilized transglutaminase was investigated relative to that of the free enzyme. The temperature profiles of free, cross-linked and immobilized transglutaminase were measured by incubating the enzyme at 30, 35, 37, 40, 45, 50, 55 and 60 °C for 10 min, respectively.

The optimum temperature of free transglutaminase was determined to be approximately 40 °C. However, cross-linking and immobilization resulted in an increase to approximately 45 °C and 50 °C, respectively, thus indicating a significant alteration of enzyme microenvironment on the polypropylene microporous membrane surface. This suggests that crosslinking and immobilization results in strengthening of the enzyme structure and provides a protective effect against heat denaturation.

**Figure 2:**
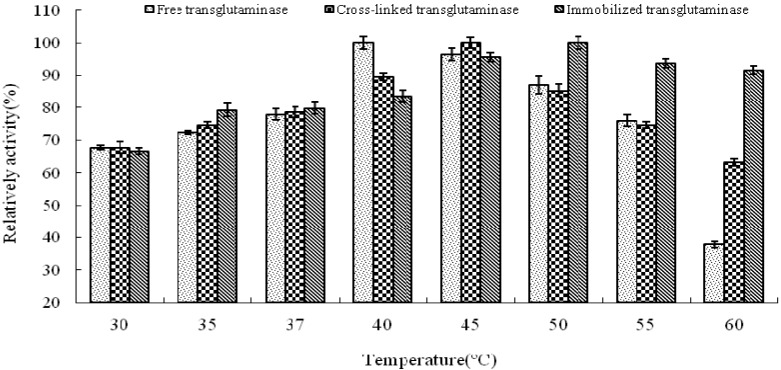
Temperature profile of free, cross-linked and immobilized transglutaminase. Data are expressed relative to the maximal enzyme activity.

The activities of the enzymes at different temperatures were expressed relative to the maximum activity of free, cross-linked and immobilized crystalline transglutaminase at the optimum temperatures of 40, 45 and 50 °C respectively (Figure 2). At 60 °C, immobilized, cross-linked and free transglutaminase retained 91.7 ± 1.20%, 63.2 ± 1.05% and 37.9 ± 0.98% maximum activity respectively.

These results indicate that the optimal temperature of transglutaminase activity was altered by the state of the enzyme and was attributed to the cross-linking treatment and immobilization. The immobilization procedure seems to protect the enzymatic configuration from distortion or damage by heat exchange and as a result; immobilized enzyme could work in tougher environment with minimal activity loss.

**Figure 3:**
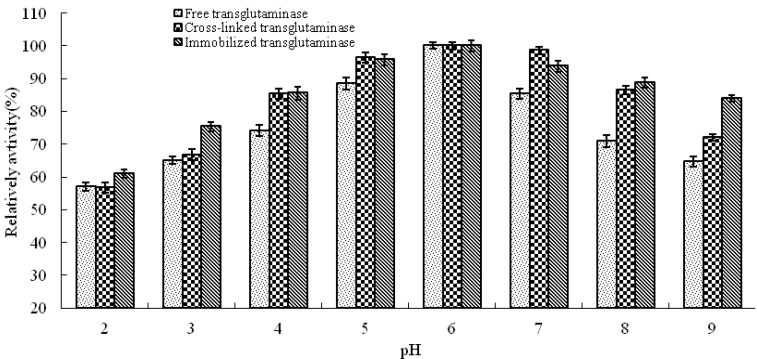
pH profile of free, cross-linked and immobilized transglutaminase. Data are expressed relative to the maximal enzyme activity.

pH is known to be a significant parameter responsible for alteration in enzymatic activity in aqueous solution. In this study, the effect of pH on the activity of free, cross-linked and immobilized transglutaminase was studied at a range of pH values at 37 °C (Figure 3). The optimum pH of free transglutaminase was determined to be 6.0 and was not altered by cross-linking or immobilization.

### 2.3. Thermal and pH Stabilities

**Figure 4:**
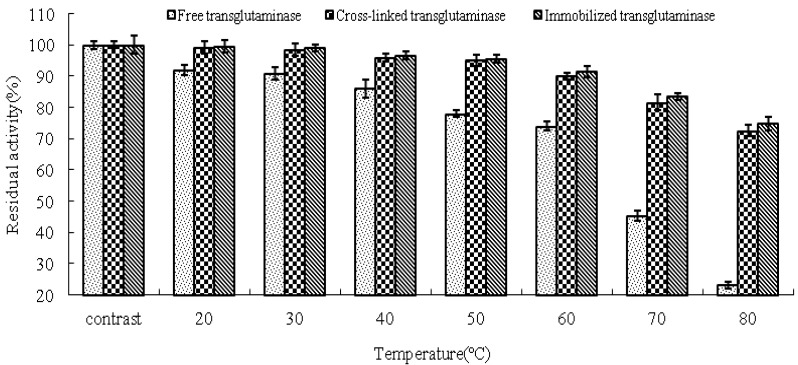
Thermal stabilities of the free, cross-linked and immobilized transglutaminase at different temperatures. Data are expressed as the % residual enzyme activity compared with the control.

The thermal stabilities of free, cross-linked and immobilized transglutaminase were determined in terms of the residual activities compared with the control (Figure 4). The activity of cross-linked and immobilized transglutaminase increased significantly in comparison to that of the free trans-glutaminase at all temperatures investigated. After 120 min incubation at 80 °C, the residual activities of free, cross-linked and immobilized transglutaminase were 23.2 ± 1.05%, 72.6 ± 1.86% and 74.8 ± 2.06%, respectively. These data demonstrate that crosslinking treatment or immobilization of transglutaminase on polypropylene microporous membrane led to a significant stabilizing effect with regard to heat inactivation.

The cross-linked and immobilized transglutaminase maintains its native conformation at elevated temperature and having lower tendency to aggregate. This is because in cross-linked enzyme crystals, the enzyme molecules are symmetrically arranged and hence their native conformation is stabilized. When an enzyme forms a crystal, a very large number of stabilizing contacts are formed between individual enzyme molecules [40]. Energy must be put into the system in order to disrupt these new contacts, so that additional energy is required to break the covalent cross-links before the cross-linked enzyme begins to dissolve and then denature. The increased thermal stability is also due to the pre-ordered arrangement of the molecules by inter- and intramolecular cross-links between the crystals, and hence the rigidity of the three-dimensional arrangement of molecules in the cross-linked enzyme crystals [41].

**Figure 5:**
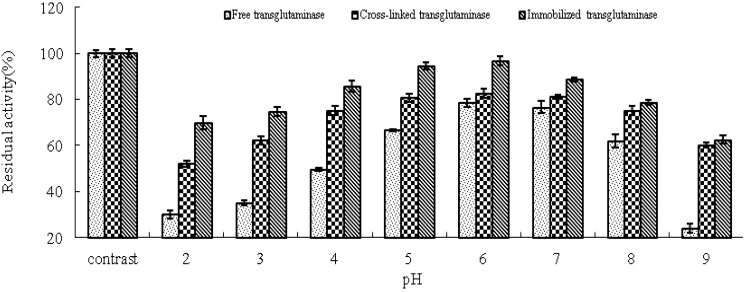
pH stabilities of free, cross-linked and immobilized transglutaminase at different pH values. Data are expressed as the % residual enzyme activity compared with the control.

The effect of pH on the residual activity of free, cross-linked and immobilized transglutaminase was studied by varying the pH of the reaction medium from pH 2.0 to pH 9.0 (Figure 5). Immobilization has been shown to result in stabilization of enzyme activity over a broad range of pH values. Under alkaline pH values (pH 8–9), free transglutaminase retained 24 ± 1.88% residual activity whereas, cross-linked and immobilized transglutaminase retained 60.1 ± 1.05% and 62.5 ± 1.62% activity, respectively. These data indicate that cross-linking treatment and immobilization provide a protective effect on enzyme activity at high pH compared with free transglutaminase.

This pH shift towards the alkaline side is due to the secondary interactions between the coupling agent and the enzyme. The change in pH stabilities depends on the charge of the enzyme and/or the matrix. The glutaraldehyde coupling of the matrix with the enzyme would have linked all the available amino groups on the surface of the enzyme, and hence the acidic groups on the enzyme surface gives a negative charge to the enzyme protein, ultimately increasing pH stabilities. The cross-linking procedure could significantly improve the enzymatic resistance against pH shift. Immobilization brought about enhanced pH stability. This may be due to the changes inflicted on the enzyme on account of the strong covalent bond formed between the support and the enzyme. Strong interactions between enzyme and support will affect the intra-molecular forces responsible for maintaining the conformation of the enzyme that would lead to a change in activity [42].

The thermal and pH stability of enzymes are very important parameters in enzyme reactor designs, as they determine the limits for use and reuse of the enzyme, and therefore process costs [43]. Cross-linked and immobilized transglutaminase have the additional advantage of good stability, and can be used in immobilized enzyme reactor to work on the polymerization and entrapment function of soybean protein. The enzyme reactor can retrieve the soy whey protein and minimize the pollution of soybean whey wastewater.

## 3. Experimental

### 3.1. Materials

Unless otherwise stated, all chemicals, reagents and solvents were of analytical grade. reduced glutathione hormone, N-α-CBZ-Gln-Gly, L-Glutamic acid γ-monohydroxamic acid were purchased from Sigma Chemical Company Ltd. Methyl methacrylate, diaminohexane and glutaraldehyde were purchased from the Tianjin Kermel Chemical Reagent Company Ltd. (Tianjin, China). Polypropylene microporous membrane (average pore diameter, 10 μm) was purchased from ZhongXin Company Ltd. (Beijing, China).

### 3.2. Enzyme Preparation

Transglutaminase derived from Streptoverticillium was obtained from Yiming Biological Products Company Ltd. (Jiangsu, China). As determined by a colorimetric hydroxamate method [44], the enzyme activity of MTGase was 9.86 U/mg of powder. The transglutaminase was purified through alcohol precipitation, ammonium sulphate precipitation, ultrafiltration and gel layer chromatography. The activity of purified enzyme was 102.8 U/mg.

### 3.3. Preparation of Transglutaminase Minicrystals

Sitting-drop vapor diffusion crystallization experiments were performed at 4 °C in 24-well Chryschem plates (type HR3-158, Hampton Research Corp., Aliso Viejo, CA, USA) sealed with Crystal Clear sealing tape (Manco Inc., Avon, OH, USA). 10 mL rarefied transglutaminase solution (15 mg/mL) was added to 10 mL 50% 2-methyl-2.4-amyldiethanol in sitting-drop trays (diameter, approx. 6 mm; maximum filling volume, approx. 40 mL). The reservoir solution contained 30 mM phosphate buffer (137 mM NaCl, 2.7 mM KCl, 4.3 mM Na_2_HPO_4_, 1.4 mM KH_2_PO_4_, pH 8.0) as the crystallization agent. After crystallization for 5 days, the crystalloid solution of transglutaminase was centrifuged at 1,000 rpm/min for 5 min. The total transglutaminase and protein content of the original solution and resulting supernatant respectively, were determined by lowry method. The transglutaminase crystal content was calculated by the following equation:

Transglutaminase crystal content (mg/mL) = total protein content of 10 mL transglutaminase solution (mg/mL) − protein content of supernatant solution (mg/mL).

### 3.4. Transglutaminase Minicrystal Cross-Linking

Transglutaminase minicrystals (50 mg) were cross-linked with 1% glutaraldehyde (pentanedial, 125 mL) at 4 °C in sodium acetate buffer (sodium acetate 54.6 g, 20 mL of 1 M acetic acid solution, diluted with deionized water to 500 mL, pH 6.0). The solution was stirred for 40 min and then filtered. The precipitate was collected and washed with deionized water several times before being washed with sodium acetate buffer (pH 6.0), until the UV value of effluent at 280 nm was below 0.5. The precipitate was lyophilized to produce the cross-linked transglutaminase crystalline powder.

### 3.5. Immobilization of Poly(methyl methacrylate)-Grafted Polypropylene Microporous Membrane

#### 3.5.1. Pretreatment of Polypropylene Microporous Membrane

Prior to grafting, polypropylene microporous membranes were pre-treated with acetone for 24 h to remove any impurities adsorbed onto the surface, dried in a vacuum oven at 30 °C to constant weight (W0) and stored in a desiccator.

#### 3.5.2. Preparation of Poly(methyl methacrylate)-Grafted Polypropylene Microporous Membranes

Poly(methyl methacrylate)-grafted polypropylene microporous membrane was prepared by UV-induced graft polymerization. Pre-treated membrane (W0) was immersed in benzophenone dissolved in acetone (0.2 mM final concentration) and simultaneously exposed to UV at a distance of 10 cm for 12 min. The membrane was dried in air, immersed in ethanol containing 20% methyl methacrylate and exposed to UV for 20 min. These reactions were performed under nitrogen gas.

Finally, the modified membrane was washed with acetone for 24 h at 30 °C with agitation to remove adsorbed monomer and homopolymer. After being dried to constant weight in a vacuum oven at 30 °C, the membrane was weighed (W) with an analytical balance to a precision of 0.1 mg. The grafting degree (GD) of poly(methyl methacrylate) was calculated by the following equation:

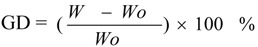

where W_0_ is the mass of the native polypropylene microporous membrane. Each result was the average of three parallel experiments.

#### 3.5.3. Introduction of the Diaminohexane Spacer Arm

Poly(methyl methacrylate)-grafted polypropylene microporous membranes were transferred to 20% diaminohexane solution. The reaction was carried out at 50 °C for 90 min. Membranes were washed under running deionized water to eliminate adsorbed diaminohexane.

#### 3.5.4. Activation of Amino Groups

The activation of the polypropylene microporous membrane amino groups was achieved by reaction with glutaraldehyde. Membranes were immersed in glutaraldehyde-water solution (2.0%, v/v, 100 mL). The reaction was carried out at 30 °C for 45 min with continuous stirring using an oscillator. Subsequently, activated membranes were removed and washed several times with deionized water before being dried in a vacuum oven for 6 h and stored at 4 °C.

#### 3.5.5. Crystalline Transglutaminase Immobilization

Immobilization of cross-linked crystalline transglutaminase onto modified poly(methyl methacrylate)-grafted polypropylene microporous membrane (3 cm × 3 cm squares) was performed by immersion in cross-linked crystalline transglutaminase solution (15 mg/mL, 10 mL) for 24 h at 4 °C. Subsequently, membranes were washed with 0.03 M sodium acetate buffer (pH 6.0) to remove unbound cross-linked crystalline transglutaminase.

#### 3.5.6. Transglutaminase Activity Assay

Transglutaminase activity was determined by hydroxamate formation from N-CBZ-Gln-Gly substrate, using the method described by Grossowicz *et al.* [42]. Substrate solution (1 mL), containing 0.2 M Tris-HCl buffer (pH 6.0, 0.4 mL), 0.1 M hydroxylamine (0.2 mL), 0.01 M reduced glutathione (0.2 mL) and 0.15 M *N*-CBZ-Gln-Gly (0.2 mL), was mixed with appropriately diluted enzyme solution (0.4 mL). The reaction mixture was incubated at 37 °C for 10 min and then stopped by adding ferric chloride trichloracetic acid reagent (0.4 mL, consisting of 1 volume 12% HCl, 1 volume 12% trichloracetic acid-TCA and 1 volume 5% ferric trichloride solution in 0.1 M HCl). After 5 min centrifugation at 10,000 g, the absorbance at 525 nm of the supernatant was measured. Enzyme activity was calibrated against a standard curve constructed using L-glutamic acid γ-monohydroxamate where one unit of transglutaminase was deﬁned as the amount of enzyme which catalyzed the formation of 1.0 µmol L-glutamic acid γ-monohydroxamate per minute at 37 °C.

### 3.6. Optimal Temperature and pH Profile

Temperature profiles of free, cross-linked and immobilized crystalline transglutaminase were measured by incubating the enzymes at 30, 35, 37, 40, 45, 50, 55 and 60 °C for 10 min. Transglutaminase activity was assayed as previously described. pH profiles of free, cross-linked and immobilized crystalline transglutaminase were measured by incubating the enzymes in buffer solutions over the pH range 2.0 to 10.0 for 10 min at the appropriate optimal temperature. Transglutaminase activity was assayed as previously described.

### 3.7. Transglutaminase Stability

The thermal stability of free, cross-linked and immobilized crystalline transglutaminase was measured in 0.2 M Tris-HCl buffer solutions adjusted to the appropriate optimum pH. Thermal stability was analyzed at 30, 40, 50, 60 and 70 °C for 120 min. After cooling, the samples were allowed to equilibrate to room temperature for estimation of residual enzyme activity at 37 °C as previously described.

The pH stability of free, cross-linked and immobilized crystalline transglutaminase was measured in 0.2 M Tris-HCl buffer solutions at the determined optimum temperature of the enzyme. pH stability was analyzed over the pH range 4.0 to 8.0 for 120 min at 40 °C. The pH of samples was then adjusted to approximately 5.5 and incubated at 37 °C for estimation of residual enzyme activity as previously described. Assay of untreated transglutaminase activity served as a control. Results are presented as the mean ± standard deviation (SD) of replicate measurements (n = 3).

### 3.8. Scanning Electron Microscopy

Scanning electron microscopy (SEM) was used to study the cross-sectional morphology of free, cross-linked and immobilized crystalline transglutaminase. Furthermore, the surface characteristics of free and cross-linked crystalline transglutaminase were compared to those of immobilized cross-linked crystalline transglutaminase and native polypropylene microporous membrane. SEM analysis was performed using a SEMS-3400 (SEMS-3400, Hitachi Instrument Ltd., Japan) operated at a 20 kV accelerating voltage.

## 4. Conclusions

Transglutaminase has become the focus of intense interest over recent years due to the potential industrial applications of the characteristic catalytic activity of this enzyme. In this study, the optimum conditions of temperature and pH for enzyme activity of free, cross-linked and immobilized transglutaminase were determined and compared. The optimum temperature for free transglutaminase activity was determined to be approximately 40 °C, while increased values of approximately 45 °C and 50 °C were determined for cross-linked and immobilized transglutaminase, respectively. However, the optimal pH of 6.0 was not altered by cross-linking treatment or immobilization. After 120 min of incubation at 80 °C, the residual activity of free transglutaminase was reduced to 23.2 ± 1.05%, while that of cross-linked and immobilized transglutaminase retained 72.6 ± 1.86% and 74.8 ± 2.06% residual activity, respectively. Free transglutaminase retained 24 ± 1.88% residual activity at pH 9.0, whereas cross-linked and immobilized transglutaminase retained 60.1 ± 1.05% and 62.5 ± 1.62% residual activity, respectively. The broad range of pH and temperatures over which transglutaminase is active following immobilization on polypropylene microporous membrane suggests the potential application of this approach in the food industry.
